# The Kernel of the Nut, or an Answer to “Proserpine”

**Published:** 1913-03

**Authors:** 


For Texas Medical Journal.

### The Kernel of the Nut, or an Answer to "Proserpine"

BY A WIFE AND MOTHER.

*"When Greek meets Greek, then comes the tug of war."*

It is indeed a pleasure to cross swords with a worthy antagonist in the arena of thought, for, "as iron sharpeneth iron," we may paraphrase, so doth interchange of thought educate us.

A good woman upon leaving church one Sunday paused at the door and took issue with her pastor on some crucial point in his sermon. Instead of becoming angry with her, he smilingly said: "Sister, I am glad the sermon did you good. I always believe I have driven a truth home somewhere, if what I say excites criticism."

So, it is with pleasure that the author of *"How to Hold Our Husbands"** welcomes Proserpine. In the first place, as the author of this now famous article maintained, it is no longer a question of "How to stop the divorce evil," or prevent its spread, but *"How to hold our husbands."*

With the agitation all over the world of woman's rights, and the clamorous demand for their adjustment, and the club mania, which I think is really soon to be catalogued with neurasthenia, "automobilitis," etc., etc., there is a proportionate decrease in the home spirit. The cares of home are more irksome than to our mothers; the love of show and display is increasing with the leisure class which society is producing, until our husbands' salaries do not clothe us any more, and, with the need of more money, our wives and mothers are becoming bread-winners too, and the vocations are being filled by the middle class; and by whist and bridge and high-five players in the "400," as their prizes are worth as much as the daily or weekly wages of the stenographer.

Hence we are confronted with a sociological situation which militates against the "old home spirit"; for women have neither the desire nor the time to cultivate husband and children as formerly, and as a result, the size of the family is being limited, and the husbands neglected, and the divorce evil is growing into a wolf, prowling at our very doors!

Along with the demand for "obey" to be stricken out of the marriage service came the feeling of intolerance all along the line, to the point of open rebellion,—Divorce.

---

*TEXAS MEDICAL JOURNAL, June and December, 1912.

The writer is glad that she said "obey," and after nearly twenty years of wearing the yoke, can still smile.

The sanctioning of the marriage relation makes it a holy ordinance; and, wives, with the words still in your ears, "whom God hath jointed together let no man put asunder," and "do you solemnly promise to take him for better, or worse, richer or poorer, until death do you part?" is just as binding, I take it, today, as it was when you said it last year—or twenty years ago.

It was in the spirit of helping the many wives, who feel that it has been "for worse," that the writer entered upon her first article, believing that as it is the woman's duty, *first to forgive,* so it is still her duty to be the first to try to find the remedy for the canker worm, eating into the heart of the flower of marriage.

In the search, let not the cataract of bitterness get in her eyes, to blind her to two facts: first, that a man does the courting before marriage, and the *woman,* afterward; and whether she has done her part. And, second, if there are children, then she owes him a debt of lasting gratitude for giving her such fine specimens (for "every crow thinks her own the blackest") and how far, or in what measure has she shown her appreciation?

"Such measure as ye mete shall be measured to you again" is still true everywhere.

Some may take issue with the writer as to whether it is *woman's duty* to be first to forgive or seek a reconciliation, but as she was made *after* Adam, so *she* has always been second in all things; and yet, was she not first at the tomb, to roll away the stone of darkness? and has she not been the leading spirit in light and truth ever since?

So she should be the first to lift the veil of misunderstanding in the home, as the role of "turtle dove," or peacemaker, best befits her nature.

Now, in the lifting of this veil of misunderstanding, she must go deep into the shadows and recesses of the rock of her fortress (the home, its life and spirit), and, with the lamp of truth in her hand, seek out the serpent that lies coiled there and *throw it out, at any cost.* Now the cost may be (as suggested in my former article) the recognizing of facts, and laying hold upon the situation bravely and boldly, and prayerfully doing as suggested, and in taking the remedy offered in the light of reason, for *mind* controls all our actions; consequently, if we can *control our minds,* we *control the whole situation.* As "the end" is believed by some to "justify the means," so to endeavor thus to turn a *loveless* relation into a *loved one,* or prostitution sanctioned by our social codes into wedded bliss, then such an attempt should not be called base, nor be said to be sacrificing principle to practice.

In upholding divorce, Proserpine quotes it as a remedy for "mismating," as "trees, fruit, etc., unfit for bearing, are cast out," etc.

The remedy for this difficulty in the human family is sterilization. For the unfit in the human, as in the plant world, should *not be permitted* to reproduce.

She further states that, "if a man is encouraged to give way" to his "one nature," asylums and early graves will continue to be filled with "sacrificed wives."

Now, in the first place, marital intercourse entered into by two normal beings is helpful, and a source of health and contentment. With the enlightenment of our day, excess in that direction is hardly known; that is, where it is mutually agreeable. If *not* mutual, then the wife has recourse to the remedy offered in the first article, although such procedure is called, by Proserpine, "base."

Now, we come back to the writer's statement that "our education was wrong, and the same propaganda is being carried on today."

We are not teaching our boys and girls what *mating* means. We should stop the false education and begin at once with our own children, and tell them that the seat of happiness is in the *sex zone* of the body. From *that* radiates all desires, etc. As it is controlled, the mind is enlarged and beautified; as it is cultivated, the soul expands; as it is abused, the body is stunted and ambition stultified.

Now, with this knowledge and instruction, our boys and girls of the future, or men and women of the great "tomorrow," should *never know divorce;* for if the girl knows that the call of her nature, the impulse of love, is to be answered by a *soul-mate,* and told to go in search of him, and *not to give* herself into the keeping of any man until she comes face to face with the one whose very presence thrills her with a "new wild joy"; whose hand clasp is as the electrode to the battery; whose voice subdues her spirit; whose eye holds her own, and whose soul calls her through space; then teach every boy not "to try" every girl, but *wait* until he suddenly hears a voice which thrills him, sees a form which possesses him with an unconquerable desire; then he will have found "the woman who offers the most to his nature," as suggested in my former article, and she will have found her "lord and master."

and "they twain shall be as one flesh," and other women or other men will not appeal to them!

Wives and mothers, hear me! Shall we be responsible for the unhappy marriages and divorces of our own children? Shall we allow prudery to wreck their lives as it has wrecked hundreds, nay, thousands, in the past? God forbid.

Mothers, listen: The matron of a rescue home told the writer, not long ago, that one of the inmates at one time was a little girl between 9 and 10 years of age, who actually gave birth to a baby, and while in labor this child-mother cried out, "Oh, he didn't tell me it was going to be a *baby;* he said he would give me a *little doll.*"

Ah, mothers, does this tragedy teach you nothing?

This same matron said she had a little 12-year-old girl inmate whose ruin was effected by a neighbor boy 14 years old, and all this in this enlightened State of ours, and in the twentieth century. What does it tell? It tells that *mothers* are not doing their duty by their girls and boys!

Ah, wives and mothers, don't tell me that you are glad your children are all boys; they are so much easier to raise. These two instances are samples of "easy" raising.

The shifting of responsibility and the simple putting of a pair of rompers on your boy, and turning him loose, ends in such tragedies as the above; for, turning him loose at that age, he will have sown his wild oats at 18, and at 23 may marry some girl whose mother was too ashamed to talk with her daughter on such subjects; and this girl, getting her ideas and knowledge from servants and schoolmates, has her mind perverted and befouled, and, holding an improper view of life, she turns into what physicians call a "sexual pervert," which is nothing more than one who is addicted to self-abuse, thus losing taste for proper married relations, and after marriage you find her in her physician's office claiming no knowledge of the sexual relation, and calling her husband "a beast," while the husband, on the other hand, may consult the same physician because his wife has no sexual desire and wants to know what to do Divorce may follow, and *you are responsible!*

Do not misunderstand me, and think that I claim that examples cited and statements made make a shoe that fits every case, but it fits so many cases that it is wise to have them pointed out, that you may keep your children's feet from slipping into them.

"When men and women are sexually made," as a wise physician has said, then "all other things shall be added unto them," for intellectual, social and physical intercourse all blend in the ideal marriage relation, and this may, with your help, be the portion of your son or your daughter. Will you make it possible for them? For—

"Queen Love, forsooth, is sacred fire
That burns within each breast,
And when her consort, King Desire,
Draws near, on holy quest,
Then there springs up a flame of light
Which sheds its radiance far,
For Faith and Hope and Charity
Now form a bright new star;
Its place is in that firmament
Whose name is Wedded Bliss,
Whose portals lead to Paradise
This side the river Styx.

